# Design-Based Approach for Analysing Survey Data in Veterinary Research

**DOI:** 10.3390/vetsci8060105

**Published:** 2021-06-08

**Authors:** D. Aaron Yang, Richard A. Laven

**Affiliations:** 1 Jockey Club College of Veterinary Medicine and Life Sciences, City University of Hong Kong, Kowloon, Hong Kong; 2 School of Veterinary Science, Massey University, Palmerston North 4442, New Zealand; r.laven@massey.ac.nz

**Keywords:** sampling, survey methodology, design-based approach, unbiasedness, variance estimation

## Abstract

Sample surveys are an essential approach used in veterinary research and investigation. A sample obtained from a well-designed sampling process along with robust data analysis can provide valuable insight into the attributes of the target population. Two approaches, design-based or model-based, can be used as inferential frameworks for analysing survey data. Compared to the model-based approach, the design-based approach is usually more straightforward and directly makes inferences about the finite target population (such as the dairy cows in a herd or dogs in a region) rather than an infinite superpopulation. In this paper, the concept of probability sampling and the design-based approach is briefly reviewed, followed by a discussion of the estimations and their justifications in the context of several different elementary sampling methods, including simple random sampling, stratified random sampling, and one-stage cluster sampling. Finally, a concrete example of a complex survey design (involving multistage sampling and stratification) is demonstrated, illustrating how finding unbiased estimators and their corresponding variance formulas for a complex survey builds on the techniques used in elementary sampling methods.

## 1. Introduction

Sample surveys, where data from a subset, or sample, of a population are used to make inferences about that population, are a traditional research methodology which has been widely used in veterinary research and investigation [1,2]. However, in this era of “big data”, with modern techniques such as machine learning, bioinformatics, or other computer-based technologies being increasingly used in veterinary research [3] across areas such as animal behaviour [4] and disease detection [5] and prediction [6], the sample survey is in danger of appearing “old fashioned” and “out-dated”.

However, the “old-fashioned” sample survey still has some advantages over cutting-edge big data methodologies. Firstly, in a sample survey, information or data can be collected actively in order to answer a specific research question, whereas the research question that can be answered by “big data” techniques is dependent on what information is available in the big data source. Secondly, in a well-planned sample survey the target population can be framed in advance and followed by a well-designed sampling process so that the samples are representative of the population [7]. This representativeness is often not achieved during the passive “big data” collection process, with data often being collected only from a particular subset of the target population—e.g., Revilla, et al. [8] analysed more than 10.5 million measurements from ~13,000 pigs obtained using automatic feeding systems. However, this dataset was collected from only one boar testing station, making generalisation to the wider population potentially difficult. Finally, it is not economically feasible to collect “big data” when novel information is required for some specific research topics.

Once the specific data for a research topic are collected, a rigorous and robust data analysis is essential to gain insight from the sample survey. As per Alexis de Tocqueville: “when statistics are not based on strictly accurate calculations, they mislead instead of guide” [9]. Generally, there are two approaches for analysing survey data: the model-based approach and the design-based approach [10]. The former is possibly better understood by many veterinary researchers who have undertaken standard quantitative research methodology training, as mainstream statistical training usually treats the observed data—e.g., production data or diagnostic test outcomes—as realisations of some relevant random variables. However, one important assumption in this method is usually overlooked—that the underlying population is treated as a “superpopulation” which contains infinitely many animals [11]. Strictly speaking, an estimated model parameter therefore refers to a property of the hypothetical “superpopulation” rather than a characteristic of the finite population which is of actual interest [7].

For example, suppose that simple random sampling had been implemented on a dairy farm to study the prevalence of bovine digital dermatitis using a large sampling fraction (~70% of the herd size). The analyst then fitted an intercept-only logistic regression to estimate the intercept. As the intercept represents the logit of the prevalence, using a suitable back-transformation, the analyst was then able to report the estimated prevalence of digital dermatitis on the farm. However, given a large sampling fraction, this estimate actually represents the prevalence of a hypothetical superpopulation from which the sample was drawn rather than the prevalence on the farm of interest [12]. To make an inference to the actual finite population, a superpopulation approach can be used [12]; however, although mathematically correct, explaining the approach is likely to create difficulties in communications with other rural professionals or companion animal practitioners.

The design-based approach for analysing survey data avoids the complexity in analysis and communication seen with the model-based approach. One key advantage of the design-based approach is that it focuses on inferences related to the target finite population(s) without introducing extra assumptions about the parametric form of the outcome variable. In addition, the analysis steps are consistent with the sampling steps, so the process of checking for potential mistakes during the analysis process is clearer [13]. Thirdly, the design-based approach has no requirement of an “assumed” probability distribution dictated by the design itself [14]. The aim of this review article is to provide a comprehensive introduction to the design-based approach for analysing survey data by (1) describing the analytical methods for elementary probability sampling methods, including simple random sampling, stratified random sampling, and cluster sampling, and (2) to demonstrate the key ideas necessary to understand and interpret those analytical methods, as well as how those ideas can be used to develop methods for any specific complex survey design.

## 2. Overview of Probability Sampling

**Figure 1:**
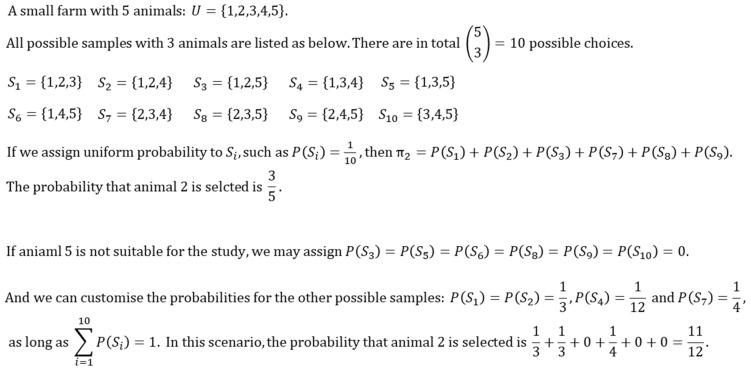
An intuitive explanation of the probability of a sample selection *P(S)* and the probability of an animal selection π.

First, we define a set *U* as a target population including *M* animals in a finite population (e.g., animals on a farm or all >2-year-old Jersey cows in a region). There are various ways to obtain samples which are just some subsets of *U*. Let us denote *S* for a particular sample chosen from *U*, then *S* ⊂ *U*. With the proper subset notation “⊂”, we restrict the sample size to being smaller than the population size. Suppose we want to obtain a random sample with *m* animals, we could have $\left(\begin{array}{c} M\\ m \end{array}\right)=L$ different samples: S_1_, S_2_, S_3_, … We can then define a set “sample space” (denoted as Ω) that contains all these samples. With probability sampling, a probability can be explicitly assigned for each of the samples, with the constraint that $\sum_{i=1}^{L}P\left(S_{i}\right)=1$, as the axiom states that the probability of a sample space is 1 and the union of all the samples forms the sample space. The probability of obtaining each of the *L* samples does not have to be constant—i.e., $P\left(S_{1}\right)\neq P\left(S_{2}\right)$ is absolutely acceptable—and we can also restrict the probability of a particular sample to 0 if some animals within the sample are considered inappropriate as study units. The other feature of these samples is that two samples can include the same animals, and the probability of an animal *k* being selected $\left(\pi_{k}\right)$ is calculated by summing the probabilities of all samples including this animal—i.e., $\pi_{k}=\sum_{S:k\in S}P\left(S\right)$. An intuitive numeric example is displayed in Figure 1. Eventually, we define the sampling weights $w_{k}$ as the reciprocal of the inclusion probability $\pi_{k}$ for any type of sampling method [15]. Generally, it is recommended that the veterinary researcher interprets the sampling weight of the animal *k* as the number of animals in the target population represented by this animal (a deeper treatment of sampling weights can be found in Gelman [16]; however, non-response adjustments are beyond the scope of this article).

## 3. Design-Based and Model-Based Approaches

### 3.1. Overview of Design-Based Approach

In the design-based approach, the observed value (production record or test outcome) $y_{k}$ is not considered to be a realisation generated from some data generation mechanism (or “population”); instead, it is regarded as a fixed constant, with the randomness arising solely from the sample selection [17]. In plainer language, although $y_{k}$ is fixed, it remains unknown unless the animal is selected in the sample [18]. If all animals are tested from a given population, then the test results for all the animals are known without any uncertainty. In contrast, if we only test a sample of these animals, the only outcomes that we know are the animals included in the sample. If we randomly draw samples of a fixed size repeatedly from a target population, a particular animal may not be sampled repeatedly due to the randomness in the sample selection; hence, the sample statistics can vary across the samples. That is the only source of randomness. Therefore, it is natural to define a Bernoulli random variable to indicate whether an animal in the target population is also in the sample. For example, if the *k*th animal is included in the sample, the Bernoulli random variable $Z_{k}=1$; otherwise, it is $Z_{k}=0$. This random variable maps the (hypothetical) animal ID in the population into numeric values for selection status. This idea is essential when studying the properties of the estimators (unbiasedness) and deriving the variances of these estimators [19].

The design-based approach is of particular value when the finite population characteristics are of interest, as when a design-based approach is used, the researcher can direct inferences about the finite target population, even if the sample size to population size ratio is not small (i.e., when the finite population correction must be considered). For example, the prevalence in a finite population is interpreted as the proportion of diseased animals in that population. Assuming that 70% of the population is sampled, a design-based approach gives a direct estimate of this population proportion, which is often a key target of veterinary investigations. In addition, the estimators (i.e., the rules or formulas) for estimating the finite population characteristics are consistent with the sampling method. Therefore, the estimation process is naturally understandable and easier to communicate with non-statistically inclined veterinarians and researchers [20]. Finally, with the design-based approach, the analyst does not need to decide which potential model generated the data, as the observed values are treated as fixed constants. For example, if the average milk production in a herd is of interest, one does not need to assume that milk yield from a cow is generated from a normal random variable, particularly when it is not. One just needs to calculate the sample mean as an estimate of the average milk production in the herd. Finally, whether an estimator is unbiased (i.e., whether its expected value and the true value of the parameter are effectively equivalent) is not dependent on the parametric form of the observed value.

### 3.2. Overview of Model-Based Approach

Although this approach may be more familiar to researchers, we do not advocate this approach in this paper from a practical point of view. As with most mainstream statistical methods, this approach treats the observed value as a realisation of an underlying random variable. For example, the test result $y_{k}$ of the *k*th animal is generated from a random variable $Y_{k}$, whose parametric form must be decided. If $Y_{k}$ is a Bernoulli random variable, then with the model-based approach a likelihood-based method is the most common approach for estimating the probability that a random animal will test positive. However, this probability is not a finite population prevalence; it is more correctly interpreted as a hypothetical infinite superpopulation prevalence. Although extra steps can help to make an inference back to the finite population, this significantly adds complexity to aspects of analysis and in communicating the results to stakeholders without a statistical background [12]. The other major disadvantage of the model-based approach is that the estimated parameters may be biased if the model is mis-specified [20].

## 4. Sampling Methods

### 4.1. Simple Random Sampling

Simple random sampling (SRS) is the most basic form of probability sampling. In this process, all possible samples of a given size have the same probability of being selected—i.e., $P\left(S\right)$ is constant for every possible sample. As a result, all the animals in the population have an equal probability of being included in the sample—i.e., the inclusion probability π is the same for every animal [21]. This sampling process has been applied to many veterinary studies, including recent investigations of lumpy skin disease [22], bovine mastitis [23], and foot-and-mouth disease [24]. Despite its simplicity, in the right situation it can be a powerful sampling method and provide the theoretical basis for more complicated sampling methods. There are two forms of SRS—with and without replacement. In this article, we will limit the discussion to SRS without replacement (the sample contains no duplicated animals,) as this is by far the most common practice in veterinary research.

The statistics in which a researcher is usually interested are the properties of the population, e.g., the average milk production of the herd or the prevalence of a disease within the herd. We denote this finite population mean as μ and prevalence p is just a special case of the finite population mean when the individual outcome value $y_{k}$ can only be 1 or 0. In the SRS setting, estimating the mean is straightforward. However, for other sampling processes this is not always the case; hence, it is easier to start to estimate the finite population total before moving on to the mean (which is a linear function of the total). To ensure a consistent methodology is used in this review, we will stick with the two-step process—estimating the total first and then the mean or prevalence.

Suppose we have a herd with *M* animals, of which a sample of *m* animals has been obtained using SRS. The Horvitz–Thompson (HT) estimator of the finite population total is [25]:

(1) $$\hat{\tau }=\sum_{k=1}^{m}w_{k}y_{k}=\frac{M}{m}\sum_{k=1}^{m}y_{k}.$$

In the SRS setting, the sampling weight $w_{k}$ is a constant as the inclusion probability is the same for every animal, such that $\pi_{k}\equiv P\left(Z_{k}=1\right)=\frac{m}{M}$ (see Appendix A for technical details), where $Z_{k}$ is the Bernoulli random variable for selection and $Z_{k}=1$ if the animal *k* is selected; otherwise, $Z_{k}=0$. The HT estimator is, by design, unbiased—i.e., its expected value is equal to the true value of the finite population characteristic [7]:

$$E\left[\hat{\tau }\right]=E\left[\sum_{k=1}^{m}w_{k}y_{k}\right]=w_{k}E\left[\sum_{k=1}^{M}y_{k}Z_{k}\right]=\frac{1}{\pi_{k}}\sum_{k=1}^{M}y_{k}E\left[Z_{k}\right]=\frac{M}{m}\sum_{k=1}^{M}y_{k}\frac{m}{M}=\sum_{k=1}^{M}y_{k}=\tau ,$$

where $E\left[⦁\right]$ is the expectation operator which takes all the possible values generated by the random variable and returns the weighted average value, so $E\left[Z_{k}\right]=1\times P\left(Z_{k}=1\right)+0\times P\left(Z_{k}=0\right)=P\left(Z_{k}=1\right)=\frac{m}{M}$.

The unbiased estimator for the mean $\bar{y}$ or the prevalence $\hat{p}$ is, therefore:

(2) $$\bar{y}\equiv \hat{p}=\frac{\hat{\tau }}{M}=\frac{1}{m}\sum_{k=1}^{m}y_{k}.$$

The proof is trivial. By observing Equation (2), we see that, in SRS, the sample mean or sample proportion is the unbiased estimator for the population mean or prevalence. This means that, for other sampling strategies, building up the sample mean from SRS will also result in an unbiased estimator if done correctly.

To derive the variance of the estimator for the mean or the prevalence, it is also easier to start with the variance of the total. The detailed derivation can be seen in Appendix B; here, we only provide the formulas for the variances. First, the variance for the estimated population total is:

(3) $$Var\left(\hat{\tau }\right)=\frac{M^{2}}{m}\left(1-\frac{m}{M}\right)\sigma^{2},$$

where $\sigma^{2}=\frac{\sum_{k=1}^{M}{\left(y_{k}-\mu \right)}^{2}}{M-1}$ is the variance of the finite population. In the special case where we estimate prevalence, we can replace μ with p with some algebra (see Appendix B), resulting in $\sigma^{2}=\frac{Mp\left(1-p\right)}{M-1}$. Therefore, the variances for $\bar{y}$ and $\hat{p}$ are given as follows:

(4) $$Var\left(\bar{y}\right)=Var\left(\frac{\hat{\tau }}{M}\right)=\frac{\sigma^{2}}{m}\left(1-\frac{m}{M}\right),$$

(5) $$Var\left(\hat{p}\right)=\left(\frac{M-m}{M-1}\right)\frac{p\left(1-p\right)}{m}.$$

However, the finite population variance depends on an unknown quantity μ or p, which we are attempting to estimate; in practice, we often replace $\sigma^{2}$ with $s^{2}$ = $\frac{\sum_{k=1}^{m}{\left(y_{k}-\bar{y}\right)}^{2}}{m-1}$, which is the sample variance (or p with $\hat{p}$). Therefore, the estimated variance for $\bar{y}$ and $\hat{p}$ is:

(6) $$\hat{Var\left(\bar{y}\right)}=\frac{s^{2}}{m}\left(1-\frac{m}{M}\right),$$

(7) $$\hat{Var\left(\hat{p}\right)}=\left(1-\frac{m}{M}\right)\frac{\hat{p}\left(1-\hat{p}\right)}{m-1},$$

where $1-\frac{m}{M}$ is usually referred to as the finite population correction factor [26].

To illustrate this process, consider an investigator who wants to estimate the prevalence of digital dermatitis in lactating cows in a dairy herd. A random sample of 100 cows is obtained from a herd of 300 cows, of which 35 sampled cows are diagnosed as diseased. These 35 cows have records $y_{k}=1$ and the remining 65 sampled cows have records $y_{k}=0$. The estimated prevalence is calculated using Equation (2), thus it is 0.35. The variance of this estimate is calculated using Equation (7). As the actual prevalence is unknown, we need to use the estimated prevalence to calculate the estimated variance: $\left(1-\frac{100}{300}\right)\frac{0.35\times \left(1-0.35\right)}{100-1}=0.0015$.

### 4.2. Stratified Random Sampling

In the stratified random sampling procedure (STRRS), the target finite population (e.g., the total number of animals within a herd) is partitioned into non-overlapping groups based on some pre-defined attributes and each of the groups is referred to as a stratum. These strata constitute the entire population; therefore, each animal belongs to a specific stratum. Within each stratum, SRS is commonly used to sample animals, and the sampling processes in the different strata are independent [27]. There is no requirement to select all strata within a population. If only some strata are of interest (e.g., only those which include lactating cows), these can be selected and strata that are not of interest can be excluded. If this approach is used, it needs to be made clear that the target population is no longer the entire finite population, but rather the population represented by the selected strata.

The finite population mean or prevalence is then estimated by pooling the information from all the strata. Like SRS, STRRS is commonly used in veterinary research, for example stratification by area. This allows the researcher to investigate prevalences and associations across a country or a region—e.g., Heayns and Baugh [28] investigated the opinions of veterinarians across the UK about serological testing to assess revaccination requirements in dogs. In this study, each county of Great Britain was considered as a stratum and 10% of the small animal veterinary practices within each stratum were randomly selected (if there were fewer than 10 practices in a county, one practice was randomly chosen to represent the county). Similarly, Atuman et al. [29] investigated dog ecology, dog bites, and rabies vaccination rates in Bauchi, Nigeria, using STRRS. They stratified Bauchi into five areas, and within each area randomly selected 10% of the streets for direct street counts and the administration of a questionnaire. However, other sources of strata are also used—e.g., as part of a randomised clinical trial of footrot treatments in Kashmir, India, Kaler, et al. [30] allocated sheep with acute footrot to one of three treatments using STRRS, with the strata being based on each sheep’s maximum footrot score. Stratification is useful to ensure that the sample includes individuals which could otherwise be missed by chance in SRS due to the limited number of individuals in their stratum. For example, at a certain period a pig farm in Hong Kong may keep few finisher pigs, but many piglets and sows are present on the farm. With SRS, it is likely that none of the finisher pigs is included in the sample, therefore one can argue that there is error in the representation of the population which could potentially dimmish the accuracy of the estimate. For this reason, it is also common to sample a fixed number of individuals in each stratum. Compared to SRS, however, extra information such as the variable used for stratification (membership) must be obtained for all sampling units.

If STRRS has been used, care is required when pooling the information from the strata in order to obtain an unbiased estimator for the finite population mean or prevalence. A “natural” estimator for the mean/prevalence might involve summing up all the observed values in the sample and dividing by the sample size (equivalent to the process of the SRS). However, this estimator is unbiased if the sample size in each of the strata is proportional to the actual size of the stratum—i.e., there has been proportional allocation (this is demonstrated in more detail in Appendix C). The more general common approach to obtain an unbiased estimator for the finite population mean or prevalence follows the two principles we have mentioned: (1) following the actual sampling process and (2) starting with the finite population total. Consider a farm with M animals. A researcher has created *J* strata based on the ages of the animals. For the *j*th stratum, there are $M_{j}$ animals, and clearly $M=\sum_{j=1}^{J}M_{j}$. Suppose that $m_{j}$ animals are sampled using SRS independently from each of the strata and that the value of the variable of interest is denoted as $y_{jk}$ for the *k*th animal in the *j*th stratum.

The unbiased estimator (using weight notation) for the finite population total:

(8) $$\hat{\tau }=\sum_{j=1}^{J}\sum_{k=1}^{m_{j}}y_{jk}w_{jk},$$

where $w_{jk}$ is the sampling weight which is the reciprocal of the inclusion probability $\pi_{jk}.$ For STRRS, this is the probability of the *k*th animal in the *j*th stratum being selected. However, writing the estimator in this form is not very intuitive, and it can be rewritten into a different formula in order to provide a more intuitive and meaningful picture for veterinary researchers. As SRS has been implemented within each of the strata, the inclusion probability $\pi_{jk}$ for the *k*th animal in the *j*th stratum is simply the sample size $m_{j}$ divided by the stratum size $M_{j}$, which leads to $w_{jk}=\frac{1}{\pi_{jk}}=\frac{M_{j}}{m_{j}}$. Now, Equation (8) can be rewritten as:

(9) $$\hat{\tau }=\sum_{j=1}^{J}M_{j}\frac{\sum_{k=1}^{m_{j}}y_{jk}}{m_{j}}.$$

This formula says that in order to estimate the finite population total, we need to first compute the mean/prevalence for each of the strata ${\bar{y}}_{j}\equiv {\hat{p}}_{j}=\frac{\sum_{k=1}^{m_{j}}y_{jk}}{m_{j}}$ using the estimator we have seen in SRS and then multiply it by the stratum size $M_{j}$ to obtain the estimated total for each stratum. We then sum up all these estimated stratum totals to obtain the estimated finite population total. This is consistent with and follows the actual sampling process, as well as producing an unbiased estimator:

$$E\left[\hat{\tau }\right]=\sum_{j=1}^{J}E\left[\frac{M_{j}}{m_{j}}\sum_{k=1}^{M_{j}}y_{jk}Z_{jk}\right]=\sum_{j=1}^{J}\frac{M_{j}}{m_{j}}\sum_{k=1}^{M_{j}}y_{jk}E\left[Z_{jk}\right]=\sum_{j=1}^{J}\frac{M_{j}}{m_{j}}\sum_{k=1}^{M_{j}}y_{jk}\frac{m_{j}}{M_{j}}=\sum_{j=1}^{J}\sum_{k=1}^{M_{j}}y_{jk}=\tau ,$$

where $Z_{jk}$ is the Bernoulli random variable for selection, representing whether the *k*th animal in the *j*th stratum is selected with an inclusion probability $\pi_{jk}$, and $E\left[Z_{jk}\right]=\pi_{jk}=\frac{m_{j}}{M_{j}}$ due to SRS. Once the estimated total is found, the estimated finite population mean or prevalence is just the total divided by the population size:

(10) $$\bar{y}\equiv \hat{p}=\frac{\sum_{j=1}^{J}\frac{M_{j}}{m_{j}}\sum_{k=1}^{m_{j}}y_{jk}}{M}.$$

Since each stratum is independently sampled, building on the SRS, the variances for $\bar{y}$ and $\hat{p}$ using STRRS are also straightforward:

(11) $$Var\left(\bar{y}\right)=\frac{1}{M^{2}}Var\left(\hat{\tau }\right)=\sum_{j=1}^{J}\frac{M_{j}{}^{2}}{M^{2}}\left(1-\frac{m_{j}}{M_{j}}\right)\frac{\sigma_{j}^{2}}{m_{j}},$$

(12) $$Var\left(\hat{p}\right)=\frac{1}{M^{2}}Var\left(\hat{\tau }\right)=\sum_{j=1}^{J}\frac{M_{j}{}^{2}}{M^{2}}\left(\frac{M_{j}-m_{j}}{M_{j}-1}\right)\frac{p_{j}\left(1-p_{j}\right)}{m_{j}},$$

where both $\sigma_{j}^{2}$ and $p_{j}$ are unknown quantities representing the population variance and prevalence in the *j*th stratum. Similar to the SRS, the estimated variances are obtained by substituting estimated quantities into the unknowns, such as:

(13) $$\hat{Var\left(\bar{y}\right)}=\sum_{j=1}^{J}\frac{M_{j}{}^{2}}{M^{2}}\left(1-\frac{m_{j}}{M_{j}}\right)\frac{s_{j}^{2}}{m_{j}},$$

(14) $$\hat{Var\left(\hat{p}\right)}=\sum_{j=1}^{J}\frac{M_{j}{}^{2}}{M^{2}}\left(1-\frac{m_{j}}{M_{j}}\right)\frac{{\hat{p}}_{j}\left(1-{\hat{p}}_{j}\right)}{m_{j}-1},$$

where $s_{j}^{2}$ is the sample variance of the *j*th stratum and the formula is given in the SRS section.

To illustrate this, consider an investigation of the seroprevalence of pseudorabies on a farm where STRRS is used. First, pigs are divided into groups based on the five production stages (strata): piglets, weaners, growers, finishers, and sows (breeding herds). The total numbers of pigs in each stratum are 30, 30, 40, 20, and 60, respectively. Within each stratum, a fixed number of pigs (10) are sampled using SRS and the numbers of infected pigs are 5, 6, 3, 2, and 7. The estimated prevalence can then be calculated using Equation (10): $\frac{\frac{30}{10}\times 5+\frac{30}{10}\times 6+\frac{40}{10}\times 3+\frac{20}{10}\times 2+\frac{60}{10}\times 7}{30+30+40+20+60}=0.506$. The variance of this prevalence estimate can then be estimated using Equation (14). This is carried out stratum by stratum; for example, for the piglets, $\frac{M_{1}{}^{2}}{M^{2}}\left(1-\frac{m_{1}}{M_{1}}\right)\frac{{\hat{p}}_{1}\left(1-{\hat{p}}_{1}\right)}{m_{1}-1}=\frac{30^{2}}{{\left(30+30+40+20+60\right)}^{2}}\times \left(1-\frac{10}{30}\right)\times \frac{5}{10}\times \left(1-\frac{5}{10}\right)÷\left(10-1\right)$. This process is then repeated for all the strata, and the estimated variance is the sum of the quantities calculated for each stratum. In the example, the final estimated variance is 0.004.

### 4.3. Cluster Sampling

In this sampling method, the animals in a finite population (animals in a herd, region, or country) are aggregated into larger sampling units: clusters. A cluster is similar to a stratum; however, the sampling process is different. In a cluster sampling procedure, a set of (*n*) clusters is sampled using SRS from a population with *N* clusters. These clusters are usually referred to as primary sampling units, and the members within each cluster as secondary sampling units. Within the primary sampling units, all secondary sampling units may be measured or observed (one-stage cluster sampling) or the secondary sampling units may be sampled using SRS (two-stage cluster sampling). The selected individuals within the selected clusters then form a sample of the finite population [26]. In contrast, in STRRS all strata of interest must be included, and SRS is usually used to sample individuals within each stratum. These different sampling strategies mean that the sources of variability in cluster sampling are different from those in STRRS. In STRRS, the variability of the estimated population mean/prevalence arises only from individual variability within a stratum. For cluster sampling, the variability of the estimated population mean/prevalence comes from one or more sources [27]. In one-stage cluster sampling, where all individuals in a selected cluster are included, the variability of the estimated population characteristic or quantity is dependent on the variability between clusters. In two-stage cluster sampling, where only a sub-sample is collected from selected clusters, the variability of the estimated population characteristic comes from two sources: the within- and between-cluster variabilities [31]. One advantage of cluster sampling is that it overcomes some of the logistics issues associated with SRS or STRRS and therefore generally requires less spending on administration and travel expenses. However, the estimates provided by cluster sampling are usually less precise than those provided by SRS, given the same sample size [27].

Cluster sampling is possibly the most widely used approach in livestock research. Usually, a farm or a herd is regarded as a cluster and a number of farms/herds are selected. This was the approach adopted by Getahun, et al. [32], who studied mastitis and antibiotic resistance patterns in dairy cows in central Ethiopia. This design treated a farm as a cluster and a number of farms were chosen using SRS; within each farm, all the dairy cows were sampled. A similar approach was later used to estimate the prevalence of bovine tuberculosis in southern Ethiopia [33]. In this study, the target population was only cows above 6 months of age, and all cows above 6 months old were included on the selected dairy farms (clusters). We list here three examples of two-stage cluster sampling in veterinary research for interested readers [34,35,36]. In the rest of this section, we will first provide insights into the estimation process for one-stage cluster sampling and do the same for a two-stage cluster sampling where STRRS instead of SRS is used at the second stage (essentially a complex sampling) with details.

#### 4.3.1. One-Stage Cluster Sampling

In one-stage cluster sampling, all animals within a farm are sampled; therefore, the farm total $\tau_{i}=\sum_{k=1}^{M_{i}}y_{ik}$ is directly measured, where $y_{ik}$ is the value of the variable of interest measured for the *k*th animal on the *i*th farm given the herd size of $M_{i}$. Common research tasks might be to estimate the farm-level and animal-level averages, such as the average milk production or average number of positive animals per farm and average milk production per cow or overall prevalence at the animal level. Suppose *n* farms are sampled from *N* farms in a region using SRS. As before, to estimate the population mean or prevalence it is always recommended to start by estimating the total. Since SRS is used for sampling clusters, the unbiased estimator for the finite population total (e.g., the number of all diseased dairy cows in a region) is straightforward and therefore given without proof:

(15) $$\hat{\tau }=\sum_{i=1}^{n}w_{i}\tau_{i}=\frac{N}{n}\sum_{i=1}^{n}\tau_{i}.$$

The variance and estimated variance for this estimator can also be straightforwardly determined by applying the theory introduced in the SRS section: $Var\left(\hat{\tau }\right)=N^{2}\left(1-\frac{n}{N}\right)\frac{\sigma_{\tau }^{2}}{n}$ and $\hat{Var\left(\hat{\tau }\right)}=N^{2}\left(1-\frac{n}{N}\right)\frac{s_{\tau }^{2}}{n}$, where $\sigma_{\tau }^{2}$ and $s_{\tau }^{2}$ are the finite population variance and sample variance (at the farm level), such that $\sigma_{\tau }^{2}=\frac{1}{N-1}\sum_{i=1}^{N}{\left(\tau_{i}-\frac{\tau }{N}\right)}^{2}$ and $s_{\tau }^{2}=\frac{1}{n-1}\sum_{i=1}^{n}{\left(\tau_{i}-\hat{\frac{\tau }{N}}\right)}^{2}$. The estimated farm-level average and its corresponding variance and estimated variance are straightforward:

(16) $${\bar{y}}_{F}=\hat{\frac{\tau }{N}}=\frac{1}{n}\sum_{i=1}^{n}\tau_{i},$$

(17) $$Var\left({\bar{y}}_{F}\right)=\left(1-\frac{n}{N}\right)\frac{\sigma_{\tau }^{2}}{n},$$

(18) $$\hat{Var\left({\bar{y}}_{F}\right)}=\left(1-\frac{n}{N}\right)\frac{s_{\tau }^{2}}{n}.$$

The total number of animals in the region is $M=\sum_{i=1}^{N}M_{i}$. Hence, the estimated average at the cow level is given by:

(19) $$\bar{y}\equiv \hat{p}=\frac{\hat{\tau }}{M}.$$

The variances and estimated variances for the cow-level average or overall prevalence are given as:

(20) $$Var\left(\bar{y}\right)\equiv Var\left(\hat{p}\right)=\frac{N^{2}}{M^{2}}\left(1-\frac{n}{N}\right)\frac{\sigma_{\tau }^{2}}{n},$$

(21) $$\hat{Var\left(\bar{y}\right)}\equiv \hat{Var\left(\hat{p}\right)}=\frac{N^{2}}{M^{2}}\left(1-\frac{n}{N}\right)\frac{s_{\tau }^{2}}{n}.$$

Note that at the farm level, we work on counts of positive animals instead of binary values even if we are estimating prevalence, therefore the variance formulas for $\bar{y}$ and $\hat{p}$ are indistinguishable.

#### 4.3.2. Two-Stage Cluster Sampling

The main purpose of this section is to illustrate the estimation process for a complex survey—i.e., how to obtain the unbiased estimators and derive their corresponding variances. Suppose there are *M* dairy cows in a region with *N* dairy herds. The herd size for herd *i* is $M_{i}$. The cows are separately managed based on a certain criterion; that is, within the *i*th herd there are *J* groups, and within each of the groups there are $M_{ij}$ cows. The groups can be treated as strata, as they are not overlapping and constitute the entire herd. A research team is interested in knowing the prevalence of a disease among cows in this region. Based on the demographic information, a two-stage cluster sampling is decided. First, *n* herds will be selected using SRS. Within each of the sampled herds, STRRS will be used to sample cows from each of the strata in each of the herds. Before going to the estimation process, we shall define some notations (Table 1).

The ultimate goal for this sample survey is to estimate p; however, as in the previous examples it is the best to start by estimating the total τ. Additionally, the computation process needs to be consistent with the actual sampling steps. Thus, we start by estimating the total diseased animals in the *j*th stratum in the *i*th herd. Within each stratum, SRS is used, therefore the estimated total can be computed based on Equation (1). The second step is to estimate the total diseased animals in the *i*th herd. Because we used STRRS, this can be achieved by adopting Equation (9). Finally, we can estimate the total number of diseased animals in the region by using Equation (15), as SRS is used to select herds. Hence, the unbiased estimated region total is computed in the following way:

(22) $$\hat{\tau }=\frac{N}{n}\sum_{i=1}^{n}\sum_{j=1}^{J}\frac{M_{ij}}{m_{ij}}\sum_{k=1}^{m_{ij}}y_{ijk}.$$

To prove that the outcome of this process is unbiased, we simplify the notation, letting ${\hat{\tau }}_{i}=\sum_{j=1}^{J}\frac{M_{ij}}{m_{ij}}\sum_{k=1}^{m_{ij}}y_{ijk}$. We know that ${\hat{\tau }}_{i}$ is unbiased (namely, $E\left[{\hat{\tau }}_{i}\right]=\tau_{i})$, because we have used STRRS. Secondly, we specify a binary indicator variable $Z_{i}\;\mathrm{that}=1$ if herd *i* is selected or 0 if it is not. Let $\pi_{i}$ denote the probability that herd *i* is selected (inclusion probability of a herd); we then have $\pi_{i}\equiv P\left(Z_{i}=1\right)=E\left[Z_{i}\right]=\frac{n}{N}$, since SRS is used for the first stage of selection (i.e., the selection of herds). Given that sampling within any herd is independent of the sampling in any other herd and that ${\hat{\tau }}_{i}$ is independent of $Z=\left(Z_{1},Z_{2},\ldots ,Z_{N}\right)$, we have:

$E\left[\hat{\tau }\right]=E\left[E\left[\hat{\tau }|Z\right]\right]=E\left[E\left[\sum_{i=1}^{n}\left.\frac{N}{n}{\hat{\tau }}_{i}\right|Z\right]\right]=E\left[E\left[\sum_{i=1}^{N}\left.\frac{N}{n}Z_{i}{\hat{\tau }}_{i}\right|Z\right]\right]$ (partition theorem for expectations)
$=E\left[\sum_{i=1}^{N}E\left[\left.\frac{N}{n}Z_{i}{\hat{\tau }}_{i}\right|Z\right]\right]$ (the conditional expectation of a sum is the sum of the conditional expectations)
$=E\left[\sum_{i=1}^{N}\frac{N}{n}E\left[\left.Z_{i}{\hat{\tau }}_{i}\right|Z\right]\right]$ (expectation is a linear operator and $\frac{N}{n}$ is a constant)
$=E\left[\sum_{i=1}^{N}\frac{N}{n}Z_{i}E\left[\left.{\hat{\tau }}_{i}\right|Z\right]\right]$ (knowing a vector means the same as knowing every element of the vector; conditional on the selection status of every herd means knowing the selection status of any herd)
$=E\left[\sum_{i=1}^{N}\frac{N}{n}Z_{i}E\left[{\hat{\tau }}_{i}\right]\right]$ (${\hat{\tau }}_{i}$ and Z are independent)
$=E\left[\sum_{i=1}^{N}\frac{N}{n}Z_{i}\tau_{i}\right]$ (unbiased estimator for stratified random sampling for each herd)
$=\sum_{i=1}^{N}\frac{N}{n}\tau_{i}E\left[Z_{i}\right]=\sum_{i=1}^{N}\frac{N}{n}\tau_{i}\frac{n}{N}=\sum_{i=1}^{N}\tau_{i}=\tau$ (linear property of expectation).

Therefore, the unbiased estimator for the overall prevalence is simply:

(23) $$\hat{p}=\frac{\hat{\tau }}{M}=\frac{N}{nM}\sum_{i=1}^{n}\sum_{j=1}^{J}\frac{M_{ij}}{m_{ij}}\sum_{k=1}^{m_{ij}}y_{ijk}.$$

In order to find the variance formula for $\hat{p}$, it is easier to start with $\hat{\tau }$. It is necessary to first identify the sources of variability. In this two-stage cluster sampling process, we have between- and within-herd variances. The variance partition formula can thus decompose the total variance into two parts: $Var\left(\hat{\tau }\right)=Var(E[\hat{\tau }|Z])+E[Var(\hat{\tau }|Z)]$, where $Var(E[\hat{\tau }|Z])$ measures the variability between herds and $E[Var(\hat{\tau }|Z)]$ measures the variability within a herd. Since SRS is implemented at the herd level, according to Equation (3), we have:

$$Var\left(E\left[\hat{\tau }|Z\right]\right)=Var\left(E\left[\left.\sum_{i=1}^{N}\frac{N}{n}Z_{i}{\hat{\tau }}_{i}\right|Z\right]\right)=Var\left(\sum_{i=1}^{N}\frac{N}{n}Z_{i}\tau_{i}\right)=N^{2}\left(1-\frac{n}{N}\right)\frac{\sigma_{\tau }^{2}}{n},$$

where $\sigma_{\tau }^{2}=\frac{1}{N-1}\sum_{i=1}^{N}{\left(\tau_{i}-\frac{\tau }{N}\right)}^{2}.$ This part of the variance is the same as that of one-stage cluster sampling, since the herd sampling procedures are exactly the same. The detailed derivation is essentially the same as the derivation of variance in SRS (see Appendix B).

For the within-herd component of the variance, $E\left[Var\left(\hat{\tau }|Z\right)\right]$, the formula inside the expectation operator, $Var\left(\hat{\tau }|Z\right)=E[{\hat{\tau }}^{2}|Z]-E{\left[\hat{\tau }|Z\right]}^{2}$ according to the conditional variance formula. The detailed mathematical derivation is available in Appendix D and Appendix E provides the statistical theorems required in this paper. Here, we only give an essential intermediate result: $E\left[Var\left(\hat{\tau }|Z\right)\right]=\frac{N}{n}\sum_{i=1}^{N}Var\left({\hat{\tau }}_{i}\right).$ Since STRRS is implemented within each herd, $Var\left({\hat{\tau }}_{i}\right)$ can be easily obtained from Equation (11) or Equation (12) depending on the nature of $y_{ijk}$. In our particular example, where $y_{ijk}$ takes a binary value (either 1 or 0), we have $Var\left({\hat{\tau }}_{i}\right)=\sum_{j=1}^{J}\frac{M_{ij}^{2}}{m_{ij}}\left(1-\frac{m_{ij}}{M_{ij}}\right)\frac{M_{ij}p_{ij}\left(1-p_{ij}\right)}{M_{ij}-1}$. Generally, $Var\left({\hat{\tau }}_{i}\right)=\sum_{j=1}^{J}\frac{M_{ij}^{2}}{m_{ij}}\left(1-\frac{m_{ij}}{M_{ij}}\right)\sigma_{ij}^{2}$.

Therefore, the general formula for the variance of $\hat{\tau }$ is given as:

(24) $$Var\left(\hat{\tau }\right)=\frac{N^{2}}{n}\left(1-\frac{n}{N}\right)\sigma_{\tau }^{2}+\frac{N}{n}\sum_{i=1}^{N}\sum_{j=1}^{J}\frac{M_{ij}^{2}}{m_{ij}}\left(1-\frac{m_{ij}}{M_{ij}}\right)\sigma_{ij}^{2},$$

where $\sigma_{ij}^{2}=\left(\frac{1}{M_{ij}-1}\right)\sum_{k=1}^{M_{ij}}{\left(y_{ijk}-\mu_{ij}\right)}^{2}$, $\mu_{ij}$ is the unknown mean of the *j*th stratum in the *i*th herd. When $y_{ijk}$ takes a binary value, the special form is given by applying the method introduced in the SRS section (see Equation (5)):

(25) $$Var\left(\hat{\tau }\right)=\frac{N^{2}}{n}\left(1-\frac{n}{N}\right)\frac{1}{N-1}\sum_{i=1}^{N}{\left(\tau_{i}-\frac{\tau }{N}\right)}^{2}+\frac{N}{n}\sum_{i=1}^{N}\sum_{j=1}^{J}\frac{M_{ij}^{2}}{m_{ij}}\left(1-\frac{m_{ij}}{M_{ij}}\right)\frac{M_{ij}p_{ij}\left(1-p_{ij}\right)}{M_{ij}-1}.$$

Again, this variance depends on some unknown quantities which we have estimated. These estimates can then be used to replace these unknown quantities, as we have done previously. Thus, the estimated variance (general form) will be:

(26) $$\hat{Var\left(\hat{\tau }\right)}=\frac{N^{2}}{n}\left(1-\frac{n}{N}\right){\hat{\sigma_{\tau }}}^{2}+\frac{N}{n}\sum_{i=1}^{n}\sum_{j=1}^{J}\frac{M_{ij}^{2}}{m_{ij}}\left(1-\frac{m_{ij}}{M_{ij}}\right)s_{ij}^{2},$$

where ${\hat{\sigma_{\tau }}}^{2}=\frac{1}{n-1}\sum_{i=1}^{n}{\left({\hat{\tau }}_{i}-\frac{\hat{\tau }}{N}\right)}^{2}$. Note the difference between ${\hat{\sigma_{\tau }}}^{2}$ and $s_{\tau }^{2}$ in the one-stage cluster sampling; $s_{ij}^{2}=\left(\frac{1}{m_{ij}-1}\right)\sum_{k=1}^{m_{ij}}{\left(y_{ijk}-{\bar{y}}_{ij}\right)}^{2}$ is the sample variance within the *j*th stratum in the *i*th herd, with ${\bar{y}}_{ij}=\frac{1}{m_{ij}}\sum_{k=1}^{m_{ij}}y_{ijk}$ being the estimated sample mean. When $y_{ijk}$ takes a binary value, the special form is given as:

(27) $$\hat{Var\left(\hat{\tau }\right)}=\frac{N^{2}}{n}\left(1-\frac{n}{N}\right)\left(\frac{1}{n-1}\right)\sum_{i=1}^{n}{\left({\hat{\tau }}_{i}-\frac{\hat{\tau }}{N}\right)}^{2}+\frac{N}{n}\sum_{i=1}^{n}\sum_{j=1}^{J}M_{ij}^{2}\left(1-\frac{m_{ij}}{M_{ij}}\right)\frac{{\hat{p}}_{ij}\left(1-{\hat{p}}_{ij}\right)}{m_{ij}-1}.$$

Finally, the variance and estimated variance for $\hat{p}$ are found simply by multiplying the results of Equations (25) and (27) by a constant $\frac{1}{M^{2}}$. The same process can be applied to find the variance and estimated variance for $\bar{y}$ when $y_{ijk}$ is not limited to binary values. A numerical illustration example in this design would be tedious to present manually; we have therefore provided the Python code for computation (see the Supplementary Materials: Python code for the two-stage cluster sampling where stratification is implemented within the clusters).

## 5. Sample Size Consideration

Although the paper has focused principally on the estimation of outcomes of interest, sample size calculations are also a critical part of the study design process. For ready-to-use sample size calculation formulas, readers are directed to Stevenson 2021 [37]. However, for a complex survey where the formula needs to be derived on a case-by-case basis, it is of value to briefly introduce the principles behind the sample size calculation. The formulas for sample size calculations are closely related to the sampling distributions of the estimators. The investigator needs to come up with an expected value for the finite population characteristic of interest and then think about how precise the estimate needs to be. The narrower the sampling distribution of an estimator, the more precise the estimate needs to be (and thus the larger the sample size). Therefore, it is natural to think what the sampling distribution is and which quantity defines the spread of the distribution.

Here, we use the SRS as an example, as the SRS serves as the theoretical basis for other more complicated sampling methods. Suppose that in the SRS for estimating prevalence in a finite herd, the sampling distribution for $\hat{p}$ is approximately normal, with mean = p and variance = $\left(\frac{M-m}{M-1}\right)\frac{p\left(1-p\right)}{m}$ (see Equation (5) of this paper for the variance formula) [27]. Note that for sample size calculations as opposed to calculating the variance of an estimator from the empirical data, we use the theoretical variance formula instead of the estimated variance formula. Clearly, the variance determines the spread of a distribution and it is a function of the sample size m; thus, this is the equation we are targeting.

The investigator then needs to specify the expected prevalence $p=p_{0}$ and think about the quantiles ($q_{\alpha /2}$ and $q_{1-\alpha /2}$) of this sampling distribution at the 1−α confidence level (usually, we set 1−α=0.95). These quantiles can be interpreted as the farthest acceptable estimates from the expected prevalence, and $q_{\alpha /2}=-q_{1-\alpha /2}$ due to the symmetry of the normal density curve. This suggests that $P\left(-q_{1-\alpha /2}\leq \hat{p}\leq q_{1-\alpha /2}\right)=1-\alpha$. Let SE denote the square root of the variance in this specific example. Standardisation gives $\frac{\hat{p}-p_{0}}{SE}\sim \;N\left(0,\;1\right)$; thus, $\pm \frac{q_{1-\alpha /2}-p_{0}}{SE}=\pm z_{1-\alpha /2}$, where $\pm z_{1-\alpha /2}$ are the quantiles of standard normal distribution that we know—for example, $\pm z_{1-\alpha /2}\approx \pm 1.96$ for 95% confidence. We thus need to find a sample size value which makes $z_{1-\alpha /2}SE$ equal to the absolute difference (d) between the farthest acceptable estimate and the expected prevalence. Now, it is just a matter of solving this equation. With a little algebra, the sample size is:

(28) $$m=\frac{z_{1-\alpha /2}^{2}Mp_{0}\left(1-p_{0}\right)}{\left(M-1\right)d^{2}+z_{1-\alpha /2}^{2}p_{0}\left(1-p_{0}\right)}.$$

## 6. Conclusions

This paper has provided a brief overview of the principles of probability sampling and the design-based approach for estimating finite population characteristics. In addition, we summarised the analytical methods for various commonly used sampling methods in detail. Instead of feeding the formulas to the readers, we have attempted to introduce and illustrate the ideas to help the readers understand, interpret, derive, and prove the unbiased estimators of the design-based samples and their corresponding variance formulas. We hope the ideas and methods presented in this paper can inspire the readers, so that the readers are encouraged to find the proper estimators and corresponding variances in their own sample surveys.

## Figures and Tables

**Table 1:** Quantities used in a two-stage cluster sampling design, where stratified random sampling is implemented in the second stage.

N	The number of dairy herds in the region.
n	The number of dairy herds in the sample.
$M_{ij}$	The number of cows in the *j*th stratum in the *i*th herd.
$m_{ij}$	The sample size in the *j*th stratum in the *i*th herd.
$M_{i}$	The number of cows in the *i*th herd (herd size for herd i), $M_{i}=\sum_{j=1}^{J}M_{ij}$.
$m_{i}$	The sample size for herd i.
M	The total number of cows in the region, $M=\sum_{i=1}^{N}M_{i}.$
$y_{ijk}$	The disease outcome (1/0) of the *k*th cow in the the *j*th stratum in the *i*th herd.
$\tau_{i}$	The total number of diseased cows in the *i*th herd, $\tau_{i}=\sum_{j=1}^{J}\sum_{k=1}^{M_{ij}}y_{ijk}$.
$p_{i}$	The herd prevalence for the *i*th herd, $p_{i}=\frac{\tau_{i}}{M_{i}}$.
τ	The total number of diseased cows in the region, $\tau =\sum_{i=1}^{N}\sum_{j=1}^{J}\sum_{k=1}^{M_{ij}}y_{ijk}$.
p	The overall prevalence in the region, $p=\frac{\tau }{M}$.

## Data Availability

No new data were created or analysed in this study. Data sharing is not applicable to this article.

## Supplementary Materials

The following are available online at https://www.mdpi.com/article/10.3390/vetsci8060105/s1, File S1: Python code for the two-stage cluster sampling where stratification is implemented within the clusters.

## Appendix A

First: we consider the simple random sampling scenario. In a population with *M* animals, let a Bernoulli random variable $Z_{k}$ denote whether animal *k* is selected. $Z_{k}=1$ if the individual *k* is selected; otherwise, $Z_{k}=0$. Let $\pi_{k}\equiv P\left(Z_{k}=1\right)$ be the inclusion probability that animal *k* is selected. Claim that $\pi_{k}\equiv P\left(Z_{k}=1\right)=\frac{m}{M}$ for a sample with a fixed sample size *m*. We shall apply the principle introduced in the section “Overview of probability sampling” to prove. For a sample with a fixed sample size *m*, there are in total $\left(\begin{array}{c} m\\ M \end{array}\right)$ ways to obtain a sample. In the simple random sampling, all possible samples of a given size have the same probability of being selected—that is, $P\left(S\right)=\frac{1}{\left(\begin{array}{c} m\\ M \end{array}\right)}=\frac{m!\left(M-m\right)!}{M!}$. The inclusion probability $\pi_{k}$ is calculated by summing the probabilities of all samples including the animal *k*—i.e., $\pi_{k}=\sum_{S:k\in S}P\left(S\right)$. There are $\left(\begin{array}{c} m-1\\ M-1 \end{array}\right)$ scenarios that a sample contains the animal *k*. As once the animal *k* is fixed, we need to choose another m − 1 animal from the rest M − 1 animals to form a sample. We then just need to add $\left(\begin{array}{c} m-1\\ M-1 \end{array}\right)$ $P\left(S\right)$s up to obtain $\pi_{k}$. Therefore, $\pi_{k}=\left(\begin{array}{c} m-1\\ M-1 \end{array}\right)\frac{m!\left(M-m\right)!}{M!}=\frac{\left(M-1\right)!}{\left(m-1\right)!\left(M-m\right)!}\frac{m!\left(M-m\right)!}{M!}=\frac{m}{M}$.

Building on the idea of the simple random sampling, the inclusion probability for stratified random sampling is easy to derive. Let $Z_{jk}\sim Bern\left(\pi_{jk}\right)$ represent whether the *k*th animal in the *j*th stratum is selected, where $\pi_{jk}$ is the inclusion probability. For the *j*th stratum, $\pi_{jk}=\frac{m_{j}}{M_{j}},$ as simple random sampling is implemented within the stratum.

For the cluster sampling, let $Z_{ik}\sim Bern\left(\pi_{ik}\right)$ represent whether the *k*th animal on the *i*th farm is selected. Let random variable $Z_{i}\sim Bern\left(\pi_{i}\right)$ represent whether the *i*th farm is selected. Given a one-stage cluster sampling scenario, if the farm is selected, all animals within the farm must be selected—namely, $P\left(Z_{ik}=1|Z_{i}=1\right)=1$. As simple random sampling is used to select farms, $\pi_{i}\equiv P\left(Z_{i}=1\right)=\frac{n}{N}.$ By the law of total probability, $\pi_{ik}\equiv P\left(Z_{ik}=1\right)=P\left(Z_{ik}=1|Z_{i}=1\right)P\left(Z_{i}=1\right)+P\left(Z_{ik}=1|Z_{i}=0\right)P\left(Z_{ik}=0\right).$ If the farm is not selected, the probability of the animal on this farm being selected is 0, namely, $P\left(Z_{ik}=1|Z_{i}=0\right)=0$, therefore $\pi_{ik}=\frac{n}{N}$. The inclusion probability for the two-stage cluster sampling is similar; the difference is $P\left(Z_{ik}=1|Z_{i}=1\right)\neq 1$, but this depends on the sampling method used within a farm.

## Appendix B

The well-known results for the variance formulas in the simple random sampling will be derived here. For a finite population with size *M*, we have $Z_{1},Z_{2},\ldots ,Z_{M}$ Bernoulli random variables representing the selection of the animals. These are identically distributed with the same marginal distribution, such that $Z_{k}\sim Bern\left(\pi_{k}\right)$; however, they are not independent, as $P\left(Z_{k}=1|Z_{l}=1\right)\neq P\left(Z_{k}=1\right)$, where k≠l. The probability of the second animal being selected depends on the first animal’s selection result. For a Bernoulli random variable, we know that $E\left[Z_{k}\right]=P\left(Z_{k}=1\right)=\pi_{k}=\frac{m}{M}$ if the sample size is *m*; $Var\left(Z_{k}\right)=\frac{m}{M}\left(1-\frac{m}{M}\right)$. Therefore, $E\left[Z_{k}^{2}\right]=Var\left(Z_{k}\right)+E{\left[Z_{k}\right]}^{2}=\frac{m}{M}$. If k≠l, the probability that both animals are selected is $P\left(Z_{k}=1,Z_{l}=1\right)=P\left(Z_{l}=1\right)P\left(Z_{k}=1|Z_{l}=1\right)=\frac{m}{M}\frac{m-1}{M-1}$, and $E\left[Z_{k}Z_{l}\right]=\sum_{Z_{k}}\sum_{Z_{l}}z_{k}z_{l}P\left(Z_{k}=z_{k},Z_{l}=z_{l}\right)=P\left(Z_{k}=1,Z_{l}=1\right)=\frac{m}{M}\frac{m-1}{M-1}$. Therefore, the covariance of $Z_{k}$ and $Z_{l}$ is computed as:

$$Cov\left(Z_{k},\;Z_{l}\right)=E\left[Z_{k}Z_{l}\right]-E\left[Z_{k}\right]E\left[Z_{l}\right]=\frac{m}{M}\frac{m-1}{M-1}-\frac{m}{M}\frac{m}{M}=-\left(1-\frac{m}{M}\right)\left(\frac{1}{M-1}\right)\frac{m}{M}.$$

Now the variance of the estimated finite population total $\hat{\tau }$ shall be derived as:

$$\begin{array}{l} Var\left(\hat{\tau }\right)=Var\left(\frac{M}{m}\sum_{k=1}^{m}y_{k}\right)=\frac{M^{2}}{m^{2}}Var\left(\sum_{k=1}^{M}y_{k}Z_{k}\right)=\frac{M^{2}}{m^{2}}Cov\left(\sum_{k=1}^{M}y_{k}Z_{k},\sum_{l=1}^{M}y_{l}Z_{l}\right)=\frac{M^{2}}{m^{2}}\sum_{k=1}^{M}\sum_{l=1}^{M}y_{k}y_{l}Cov\left(Z_{k},\;Z_{l}\right)\\ =\frac{M^{2}}{m^{2}}\left\{\sum_{k=1}^{M}y_{k}^{2}Var\left(Z_{k}\right)+\sum_{k=1}^{M}\sum_{l\neq k}^{M}y_{k}y_{l}Cov\left(Z_{k},\;Z_{l}\right)\right\}\\ =\frac{M^{2}}{m^{2}}\left\{\sum_{k=1}^{M}y_{k}^{2}\frac{m}{M}\left(1-\frac{m}{M}\right)-\sum_{k=1}^{M}\sum_{l\neq k}^{M}y_{k}y_{l}\frac{m}{M}\left(1-\frac{m}{M}\right)\left(\frac{1}{M-1}\right)\right\}\\ =\frac{M^{2}}{m^{2}}\frac{m}{M}\left(1-\frac{m}{M}\right)\left(\frac{1}{M-1}\right)\left\{\left(M-1\right)\sum_{k=1}^{M}y_{k}^{2}-\sum_{k=1}^{M}\sum_{l\neq k}^{M}y_{k}y_{l}\right\}\\ =\frac{M}{m}\left(1-\frac{m}{M}\right)\left(\frac{1}{M-1}\right)\left\{\left(M-1\right)\sum_{k=1}^{M}y_{k}^{2}-\left[{\left(\sum_{k=1}^{M}y_{k}\right)}^{2}-\sum_{k=1}^{M}y_{k}^{2}\right]\right\}=\frac{M}{m}\left(1-\frac{m}{M}\right)\left(\frac{1}{M-1}\right)\left\{M\sum_{k=1}^{M}y_{k}^{2}-{\left(\sum_{k=1}^{M}y_{k}\right)}^{2}\right\}\\ =\frac{M^{2}}{m}\left(1-\frac{m}{M}\right)\left(\frac{1}{M-1}\right)\left\{\sum_{k=1}^{M}y_{k}^{2}-\frac{{\left(\sum_{k=1}^{M}y_{k}\right)}^{2}}{M}\right\}\\ =\frac{M^{2}}{m}\left(1-\frac{m}{M}\right)\left(\frac{1}{M-1}\right)\left\{\sum_{k=1}^{M}y_{k}^{2}-M{\left(\frac{\sum_{k=1}^{M}y_{k}}{M}\right)}^{2}\right\}=\frac{M^{2}}{m}\left(1-\frac{m}{M}\right)\left(\frac{1}{M-1}\right)\left\{\sum_{k=1}^{M}y_{k}^{2}-M\mu^{2}\right\}==\frac{M^{2}}{m}\left(1-\frac{m}{M}\right)\left(\frac{1}{M-1}\right)\left\{\sum_{k=1}^{M}y_{k}^{2}-2M\mu^{2}+M\mu^{2}\right\}\\ =\frac{M^{2}}{m}\left(1-\frac{m}{M}\right)\left(\frac{1}{M-1}\right)\left\{\sum_{k=1}^{M}y_{k}^{2}-2\mu M\mu +M\mu^{2}\right\}=\frac{M^{2}}{m}\left(1-\frac{m}{M}\right)\left(\frac{1}{M-1}\right)\left\{\sum_{k=1}^{M}y_{k}^{2}-2\mu \sum_{k=1}^{M}y_{k}+\sum_{k=1}^{M}\mu^{2}\right\}\\ =\frac{M^{2}}{m}\left(1-\frac{m}{M}\right)\left(\frac{1}{M-1}\right)\left\{\sum_{k=1}^{M}y_{k}^{2}-2\mu y_{k}+\mu^{2}\right\}=\frac{M^{2}}{m}\left(1-\frac{m}{M}\right)\left(\frac{1}{M-1}\right)\left\{\sum_{k=1}^{M}{\left(y_{k}-\mu \right)}^{2}\right\}. \end{array}$$

Observe that $\frac{\sum_{k=1}^{M}{\left(y_{k}-\mu \right)}^{2}}{M-1}$ is the finite population variance $\sigma^{2}$, therefore:

$$Var\left(\hat{\tau }\right)=\frac{M^{2}}{m}\left(1-\frac{m}{M}\right)\sigma^{2},$$

which is the Equation (3) shown in the main body of the text. In our particular case, $y_{k}$ is binary valued, we shall let μ≡p and $\bar{y}\equiv \hat{p}$, therefore:

$$\sigma^{2}=\frac{1}{M-1}\sum_{k=1}^{M}{\left(y_{k}-p\right)}^{2}=\frac{1}{M-1}\sum_{k=1}^{M}\left(y_{k}^{2}-2py_{k}+p^{2}\right)=\frac{1}{M-1}\sum_{k=1}^{M}y_{k}^{2}-2p\sum_{k=1}^{M}y_{k}+\sum_{k=1}^{M}p^{2}=\frac{Mp-2pMp+Mp^{2}}{M-1}=\frac{M\left(p-p^{2}\right)}{M-1}=\frac{Mp\left(1-p\right)}{M-1}.$$

and the variance of the estimated finite population prevalence are:

$$Var\left(\hat{p}\right)\equiv Var\left(\bar{y}\right)=\frac{1}{M^{2}}Var\left(\hat{\tau }\right)=\frac{1}{M^{2}}\frac{M^{2}}{m}\left(1-\frac{m}{M}\right)\sigma^{2}=\left(1-\frac{m}{M}\right)\frac{1}{m}\frac{Mp\left(1-p\right)}{M-1}=\left(\frac{M-m}{M}\right)\frac{1}{m}\frac{Mp\left(1-p\right)}{M-1}=\left(\frac{M-m}{M-1}\right)\frac{p\left(1-p\right)}{m},$$

which is Equation (5) shown in the main body of the text. Since the variance depends on the unknown quantity p, we shall replace the finite population variance $\sigma^{2}$ with the sample variance $s^{2}$ = $\frac{\sum_{k=1}^{m}{\left(y_{k}-\hat{p}\right)}^{2}}{m-1}$. With the same algebra, $s^{2}=\frac{m\hat{p}\left(1-\hat{p}\right)}{m-1}$. Therefore, the estimated variance is:

$$\hat{Var\left(\hat{p}\right)}=\frac{1}{M^{2}}\hat{Var\left(\hat{\tau }\right)}=\frac{1}{M^{2}}\frac{M^{2}}{m}\left(1-\frac{m}{M}\right)s^{2}=\frac{1}{m}\left(1-\frac{m}{M}\right)\frac{m\hat{p}\left(1-\hat{p}\right)}{m-1}=\left(1-\frac{m}{M}\right)\frac{\hat{p}\left(1-\hat{p}\right)}{m-1},$$

which is the Equation (7) shown in the main body of the text.

## Appendix C

In the stratified random sampling section, we mentioned a “natural” estimator for the prevalence on a farm—that is:

$$\overset{ˇ}{p}=\frac{1}{m}\sum_{j=1}^{J}\sum_{k=1}^{m_{j}}y_{jk}.$$

This sums up all the observed values in the sample and divides by the sample size. This estimator is generally biased; to see why, let us suppose that we sampled the entire herd (herd size = *M*):

$$p=\frac{1}{M}\sum_{j=1}^{J}\sum_{k=1}^{M_{j}}y_{jk}=\frac{1}{M}\sum_{j=1}^{J}M_{j}p_{j}\;E\left[\overset{ˇ}{p}\right]=\frac{1}{m}\sum_{j=1}^{J}E\left[\sum_{k=1}^{M_{j}}Z_{ij}y_{jk}\right]=\frac{1}{m}\sum_{j=1}^{J}\sum_{k=1}^{M_{j}}y_{jk}E\left[Z_{jk}\right]\;=\frac{1}{m}\sum_{j=1}^{J}M_{j}p_{j}\frac{m_{j}}{M_{j}}=\frac{1}{m}\sum_{j=1}^{J}m_{j}p_{j}\neq \frac{1}{M}\sum_{j=1}^{J}M_{j}p_{j}=p\;∴\;E\left[\overset{ˇ}{p}\right]-p\neq 0\;⟹\overset{ˇ}{p}\;\mathrm{is}\;a\;\mathrm{biased}\;\mathrm{estimator}.$$

However, under the proportional allocation, the sample size in each of the strata is taken in proportion to the actual size of the corresponding stratum, that is $m_{j}=CM_{j}$, then $\sum_{j=1}^{J}m_{j}=C\sum_{j=1}^{J}M_{j}⟹m=CM$. With a little algebra, we can see that $\frac{m_{j}}{m}=\frac{M_{j}}{M}$; therefore, $E\left[\overset{ˇ}{p}\right]=p$.

## Appendix D

In the two-stage cluster sampling, the variance of the estimated total comes from two sources. Based on the variance partition formula, $Var\left(\hat{\tau }\right)=Var(E[\hat{\tau }|Z])+E[Var(\hat{\tau }|Z)]$, where $Var(E[\hat{\tau }|Z])$ measures the variability between herds and $E[Var(\hat{\tau }|Z)]$ measures the variability within a herd. Since simple random sampling is implemented at the herd level, $Var(E[\hat{\tau }|Z])$ is relatively easier to derive by following the method shown in the simple random sampling scenario.

Here, we focus on deriving the variance formula for the within-herd component $E\left[Var\left(\hat{\tau }|Z\right)\right]$. We apply the conditional variance formula inside the expectation operator: $E\left[Var\left(\hat{\tau }|Z\right)\right]=E\left[E[{\hat{\tau }}^{2}|Z]-E{\left[\hat{\tau }|Z\right]}^{2}\right]$. We will work on the two terms inside the expectation operator, then apply the expectation. A gentle courtesy here is to explain the notations again. A Bernoulli random variable $Z_{i}\sim Bern(\pi_{i})$ represents whether the herd *i* is selected, and we have $\pi_{i}\equiv P\left(Z_{i}=1\right)=E\left[Z_{i}\right]=E\left[Z_{i}^{2}\right]=\frac{n}{N}$. Sampling within any herd is independent of the sampling in any other herd and ${\hat{\tau }}_{i}$ is independent of $Z=\left(Z_{1},Z_{2},\ldots ,Z_{N}\right)$.

$$\begin{array}{l} Var\left(\hat{\tau }|Z\right)=E[{\hat{\tau }}^{2}|Z]-E{\left[\hat{\tau }|Z\right]}^{2}=E\left[\left.{\left(\sum_{i=1}^{N}\frac{N}{n}Z_{i}{\hat{\tau }}_{i}\right)}^{2}\right|Z\right]-{\left(\sum_{i=1}^{N}\frac{N}{n}Z_{i}\tau_{i}\right)}^{2}\\ =E\left[\left.\sum_{i=1}^{N}\frac{N^{2}}{n^{2}}Z_{i}{}^{2}{\hat{\tau }}_{i}{}^{2}+\sum_{i=1}^{N}\sum_{h\neq i}^{N}\frac{N^{2}}{n^{2}}{\hat{\tau }}_{i}{\hat{\tau }}_{h}Z_{i}Z_{h}\right|Z\right]-{\left(\sum_{i=1}^{N}\frac{N}{n}Z_{i}\tau_{i}\right)}^{2}\\ =\frac{N^{2}}{n^{2}}\sum_{i=1}^{N}E\left[\left.Z_{i}{}^{2}{\hat{\tau }}_{i}{}^{2}\right|Z\right]+\frac{N^{2}}{n^{2}}\sum_{i=1}^{N}\sum_{h\neq i}^{N}E\left[{\hat{\tau }}_{i}{\hat{\tau }}_{h}Z_{i}Z_{h}|Z\right]-{\left(\sum_{i=1}^{N}\frac{N}{n}Z_{i}\tau_{i}\right)}^{2}\\ =\frac{N^{2}}{n^{2}}\left(\sum_{i=1}^{N}Z_{i}{}^{2}E\left[\left.{\hat{\tau }}_{i}{}^{2}\right|Z\right]+\sum_{i=1}^{N}\sum_{h\neq i}^{N}Z_{i}Z_{h}E\left[{\hat{\tau }}_{i}{\hat{\tau }}_{h}|Z\right]-\sum_{i=1}^{N}Z_{i}{}^{2}\tau_{i}{}^{2}-\sum_{i=1}^{N}\sum_{h\neq i}^{N}Z_{i}Z_{h}\tau_{i}\tau_{h}\right)\\ =\frac{N^{2}}{n^{2}}\left(\sum_{i=1}^{N}Z_{i}{}^{2}E\left[{\hat{\tau }}_{i}{}^{2}\right]+\sum_{i=1}^{N}\sum_{h\neq i}^{N}Z_{i}Z_{h}E\left[{\hat{\tau }}_{i}{\hat{\tau }}_{h}\right]-\sum_{i=1}^{N}Z_{i}{}^{2}\tau_{i}{}^{2}-\sum_{i=1}^{N}\sum_{h\neq i}^{N}Z_{i}Z_{h}\tau_{i}\tau_{h}\right) \end{array}$$

(${\hat{\tau }}_{i}$ and Z are independent random variables, real valued functions *f* and *g* defined for ${\hat{\tau }}_{i}$ and Z are also independent random variables; this justifies $E\left[\left.{\hat{\tau }}_{i}{}^{2}\right|Z\right]=E\left[{\hat{\tau }}_{i}{}^{2}\right]$ as *f* and *g* are a quadratic and an identity functions, respectively; more generally ${\hat{\tau }}_{i}$, ${\hat{\tau }}_{h}$ and Z are independent, thus *f*(${\hat{\tau }}_{i},{\hat{\tau }}_{h}$) and *g*(Z) are independent; this justifies $E\left[{\hat{\tau }}_{i}{\hat{\tau }}_{h}|Z\right]=$ $E\left[{\hat{\tau }}_{i}{\hat{\tau }}_{h}\right]$ as *f*(${\hat{\tau }}_{i},{\hat{\tau }}_{h}$) = ${\hat{\tau }}_{i}{\hat{\tau }}_{h}$ and *g*(Z) = Z)

$$=\frac{N^{2}}{n^{2}}\left(\sum_{i=1}^{N}Z_{i}{}^{2}E\left[{\hat{\tau }}_{i}{}^{2}\right]+\sum_{i=1}^{N}\sum_{h\neq i}^{N}Z_{i}Z_{h}E\left[{\hat{\tau }}_{i}\right]E\left[{\hat{\tau }}_{h}\right]-\sum_{i=1}^{N}Z_{i}{}^{2}\tau_{i}{}^{2}-\sum_{i=1}^{N}\sum_{h\neq i}^{N}Z_{i}Z_{h}\tau_{i}\tau_{h}\right)$$

(sampling independently from the farms implies independence between ${\hat{\tau }}_{i}$ and ${\hat{\tau }}_{h}$, for two independent random variables, $E\left[{\hat{\tau }}_{i}{\hat{\tau }}_{h}\right]=E\left[{\hat{\tau }}_{i}\right]E\left[{\hat{\tau }}_{h}\right]$)

$$\begin{array}{l} =\frac{N^{2}}{n^{2}}\left(\sum_{i=1}^{N}Z_{i}{}^{2}\left\{E{\left[{\hat{\tau }}_{i}\right]}^{2}+Var\left({\hat{\tau }}_{i}\right)\right\}+\sum_{i=1}^{N}\sum_{h\neq i}^{N}Z_{i}Z_{h}\tau_{i}\tau_{h}-\sum_{i=1}^{N}Z_{i}{}^{2}\tau_{i}{}^{2}-\sum_{i=1}^{N}\sum_{h\neq i}^{N}Z_{i}Z_{h}\tau_{i}\tau_{h}\right)\\ =\frac{N^{2}}{n^{2}}\left(\sum_{i=1}^{N}Z_{i}{}^{2}\tau_{i}{}^{2}+\sum_{i=1}^{N}Z_{i}{}^{2}Var\left({\hat{\tau }}_{i}\right)-\sum_{i=1}^{N}Z_{i}{}^{2}\tau_{i}{}^{2}\right)=\frac{N^{2}}{n^{2}}\sum_{i=1}^{N}Z_{i}{}^{2}Var\left({\hat{\tau }}_{i}\right).\\ ∴\;E\left[Var\left(\hat{\tau }|Z\right)\right]=E\left[\frac{N^{2}}{n^{2}}\sum_{i=1}^{N}Z_{i}{}^{2}Var\left({\hat{\tau }}_{i}\right)\right]=\frac{N^{2}}{n^{2}}\sum_{i=1}^{N}E\left[Z_{i}{}^{2}\right]Var\left({\hat{\tau }}_{i}\right)=\frac{N}{n}\sum_{i=1}^{N}Var\left({\hat{\tau }}_{i}\right) \end{array}$$

(sampling independently from the farms).

## Appendix E

1. Joint probability: $P\left(A,B\right)=P\left(A|B\right)P\left(B\right)$,
2. Independence of two events: $P\left(A|B\right)=P\left(A\right),$
3. Law of total probability: $P\left(A\right)=P\left(A|B\right)P\left(B\right)+P\left(A|B^{c}\right)P\left(B^{c}\right),$
4. Expectation of a discrete random variable: $E\left[X\right]=\sum_{x}xf\left(x\right),$
5. Property of expectation: $E\left[aX+b\right]=aE\left[X\right]+b,$
6. Variance of a random variable: $Var\left(X\right)=E\left[X^{2}\right]-E{\left[X\right]}^{2}$
7. Property of variance:
  - $Var\left(aX+b\right)=a^{2}Var\left(X\right),$
  - If $X_{1},X_{2},\ldots ,X_{n}$ are mutually independent, then $Var\left(\sum_{i=i}^{n}X_{i}\right)=\sum_{i=i}^{n}Var(X_{i}),$
8. Bias: $\mathrm{Bias}\left(\hat{\theta }\right)=E\left[\hat{\theta }\right]-\theta ,$ unbiasedness implies $\mathrm{Bias}\left(\hat{\theta }\right)=0,$
9. Expectation for a function of two random variables: $E\left[g\left(X,Y\right)\right]=\sum_{x}\sum_{y}g\left(x,y\right)f\left(x,y\right),$
10. Covariance: $Cov\left(X,Y\right)=E\left[XY\right]-E\left[X\right]E\left[Y\right],$
11. Properties of covariance:
  - Independence between X and $Y⟹Cov\left(X,Y\right)=E\left[XY\right]-E\left[X\right]E\left[Y\right]=0,$
  - $Var\left(X\right)=Cov\left(X,X\right),$
  - $Cov\left(aX+b,cX+d\right)=acCov\left(X,Y\right),$
  - $Cov\left(\sum_{i=1}^{n}X_{i},\sum_{j=1}^{n}X_{j}\right)=\sum_{i=1}^{n}\sum_{j=1}^{n}Cov\left(X_{i},X_{j}\right)=\sum_{i=1}^{n}Var\left(X_{i}\right)+\sum_{i=1}^{n}\sum_{j\neq i}^{n}Cov\left(X_{i},X_{j}\right),$
12. Conditional expectation: $E\left[X|Y\right]$ is a random variable subject to the variation of X,
13. Properties of conditional expectation:
  - Independence between X and $Y⟹E\left[X|Y\right]=E\left[X\right]$ and $E\left[g\left(X\right)|Y\right]=E\left[g\left(X\right)\right],$
  - $E\left[g\left(Y\right)|Y\right]=g\left(Y\right)$ and $E\left[g\left(Y\right)X|Y\right]=g\left(Y\right)E\left[X|Y\right],$
  - $E\left[aX+bY|Z\right]=aE\left[X|Z\right]+bE\left[Y|Z\right],$
14. Partition theorem for expectations: $E\left[X\right]=E\left[E\left[X|Y\right]\right],$
15. Variance partition formula: $Var\left(X\right)=Var\left(E\left[X|Y\right]\right)+E\left[Var\left(X|Y\right)\right],$
16. Conditional variance formula: $Var\left(X|Y\right)=E\left[X^{2}|Y\right]-E{\left[X|Y\right]}^{2}.$
