# Supplementary material for: Design-Based Approach for Analysing Survey Data in Veterinary Research
Source: Vet Sci. 2021 Jun 8;8(6):105. doi: 10.3390/vetsci8060105 (PMC8227077; doi:10.3390/vetsci8060105)
Supplement: Supplementary file 1 [file vetsci-08-00105-s001.zip › vetsci-1195400-supplementary.pdf]

In [1]:
```
import pandas as pd
import numpy as np
from scipy.stats import norm
```

In [2]:
```
def psu_strata_prev(df,dis_name,psu,strata):
    pij_dict = {}

    for i in range(1,len(df[psu].unique())+1):
        for j in range(1,len(df[strata].unique())+1):
            t_ij = sum(df[dis_name] [ (df[psu]==i) & (df[strata] == j)])
            p_ij = t_ij / len(df[dis_name] [ (df[psu]==i) & (df[strata] == j)])
            pij_dict.setdefault(i, []).append(p_ij)

    return pij_dict
```

In [3]:
```
def psu_strata_total(df,dic):
    tij_dict = {}

    for i in range(len(df)):
        for j in range(len(df.columns)):
            tij_dict.setdefault(i+1,[]).append(df.iloc[i,j] * dic[i+1][j])

    return tij_dict
```

In [4]:
```
def psu_total(dic):
    t_i = []

    for key,value in dic.items():
        t_i.append(sum(value))

    return t_i
```

In [5]:
```
def region_total(a_list,df_pop,n_psu):
    t = sum(a_list) * len(df_pop) / n_psu
    return t
```

In [6]:
```
def region_prev(df,val):
    m_sum = 0
    for i in range(len(df)):
        for j in range(len(df.columns)):
            m_sum += df.iloc[i,j]

    p = val / m_sum

    return p
```

In [7]:
```
def overall_prev(df_sample,df_pop,dis_name,psu,strata):
    n = len(df_sample[psu].unique())
    sub_df_pop = df_pop.iloc[:n,]
    p_ij = psu_strata_prev(df_sample,dis_name,psu,strata)
    t_ij = psu_strata_total(sub_df_pop,p_ij)
    t_i = psu_total(t_ij)
    t = region_total(t_i,df_pop,n)
    p = region_prev(df_pop,t)
    return p
```

In [8]:
```
def between_var(df_sample,df_pop,dis_name,psu,strata):
    n = len(df_sample[psu].unique())
    N = len(df_pop)
    sub_df_pop = df_pop.iloc[:n,]
    p_ij = psu_strata_prev(df_sample,dis_name,psu,strata)
    t_ij = psu_strata_total(sub_df_pop,p_ij)
    t_i = psu_total(t_ij)
    t = region_total(t_i,df_pop,n)
    var_btw = sum((t_i - t / N)**2) * ((N**2) / n) * (1 - n/ N) * (1 / (n - 1))
    return var_btw
```

In [9]:
```
def within_var(df_sample, df_pop, dis_name, psu, strata):

    n = len(df_sample[psu].unique())
    N = len(df_pop)
    sub_df_pop = df_pop.iloc[:n,]
    M_ij_array = np.array(sub_df_pop)

    p_ij = psu_strata_prev(df_sample,dis_name,psu,strata)
    p_ij_array = np.array(list(p_ij.values()))

    m_ij_dict = {}
    for i in range(1,len(df_sample[psu].unique())+1):
        for j in range(1,len(df_sample[strata].unique())+1):
            m_ij = len(df_sample[dis_name] [ (df_sample[psu]==i) & (df_sample[strata] == j)])
            m_ij_dict.setdefault(i, []).append(m_ij)
    m_ij_array = np.array(list(m_ij_dict.values()))

    array_cal = (1 - m_ij_array / M_ij_array) * ((M_ij_array)**2) * ((p_ij_array * (1 - p_ij_array)) / (m_ij_array - 1))

    var_wt = np.sum(np.sum(array_cal, axis=1)) * N / n

    return var_wt
```

In [10]:
```
def var_prev(df_sample, df_pop, dis_name, psu, strata):
    var_w = within_var(df_sample, df_pop, dis_name, psu, strata)
    var_b = between_var(df_sample,df_pop,dis_name,psu,strata)
    var_t = var_b + var_w
    M = np.sum(np.array(df_pop))
    var_p = var_t / (M**2)
    return var_p
```

In [11]:
```
def CI_norm_approx(df_sample, df_pop, dis_name, psu, strata,conf_lv):
    var = var_prev(df_sample, df_pop, dis_name, psu, strata)
    mean = overall_prev(df_sample,df_pop,dis_name,psu,strata)
    SE = np.sqrt(var)
    alpha = 1 - conf_lv
    upper = mean + norm.ppf((1 - alpha / 2))*SE
    lower = mean - norm.ppf((1 - alpha / 2))*SE
    return lower, upper
```
